# Observations on the Treatment of Gun-Shot Wounds

**Published:** 1790

**Authors:** Robert Jackson

**Affiliations:** Physician at Stockton, in the County of Durham


VIII. *Obſervations on the Treatment of Gun-ſhot Wounds. Communicated in a Letter to Dr.* Simmons *by* Robert Jackſon, *M. D. Phyſician at Stockton, in the County of Durham.*

IF the following obſervations on the treatment of gun-ſhot wounds ſhould appear to be of ſuch importance as to deſerve the notice of the Public, I ſhall beg the favour of you to inſert them in the London Medical Journal.

I am aware that the cuſtom of dilating wounds made by fire arms is ſo generally adopted by practitioners, and ſo formidably ſupported by great authorities, that it requires no ſmall ſhare of courage to raiſe doubts concerning its propriety, or even its neceſſity. I am alſo aware that I am ill qualified to illuſtrate the ſubject in a regular and ſyſtematic diſcuſſion. I have little acquaintance with ſurgical writers, and cannot boaſt a very extenſive range of experience: but I hope to be indulged with the liberty of relating a few facts which occurred to me during the late war in America, and which ſeem to prove that the cuſtomary dilatation of wounds may be frequently omitted, not only without detriment, but even with obvious benefit to the patient.

In the year 1779 a party of the Loyal American militia, who had attacked a poſt of the enemy in the back parts of the province of Georgia, were obliged to retire without effecting their purpoſe. A conſiderable number of them were wounded; and as their diſtance from the army precluded them from ſurgical aſſiſtance, their wounds were only bound over with a bit of rag. Such was the fact: the conſequence was not what might have been expected; for among the number of wounds which I afterwards ſaw there were ſeveral which we ſhould have expected to be troubleſome and tedious in cure, if treated according to the common rules of ſurgery, but in reality they healed ſpeedily and well. Another action was fought, ſome time after, at Briar Creek. The greater number of the wounded were conveyed to the hoſpital, and furniſhed with proper accommodations and ſurgical aſſiſtance; but there were alſo ſome of the militia who remained in the woods, and paid no other attention to their wounds than ſimply to bind them up. The contraſt in point of treatment was obvious in the preſent caſe, and the difference of effect was not leſs remarkable; for in as far as I could judge, from thoſe examples which I had an opportunity of comparing, the progreſs towards healing was not leſs advanced in three weeks, where there was not any thing done, than in five, where art and ſkill were employed. There occurred likewiſe ſeveral accidental inſtances during the following campaigns, where the uſual formality of ſurgical treatment did not appear to be attended with conſpicuous advantage; but the faireſt comparative trial which has yet fallen under my obſervation was after the action at Cowpenn, in South Carolina, in January, 1781. The ſcene of this action was near the mountains, in a diſtrict of country almoſt uninhabited. Not fewer than an hundred and twenty men lay wounded on the field, or diſperſed themſelves in the neighbourhood, where they accidentally found the ſhelter of a hut. As I was the only profeſſional perſon who remained on the ſpot after the defeat, it was not in my power to viſit every one; neither did I find out, till after ſeveral days, the abodes to which many had retired. I may obſerve, that I dilated freely, and treated according to the uſual methods of ſurgeons, the wounds of ſuch as I ſaw early; but there was probably near near a fifth of the whole to whom, from their remote ſituation, immediate help could not be given. Other ſurgeons were afterwards ſent from the army by Lord Cornwallis, and ſuch accommodations were provided for the wounded as a deſolate country could afford. I ſtill continued my aſſiſtance, and remained on this duty near ſix weeks, during which time I had the opportunity of obſerving, that thoſe wounds to which little or nothing had been done generally healed more rapidly, and were ſeldom attended with ſo much pain and inflammation as where dilatation, poulticing, &c. had been freely employed.

It appears, then, that we may ſafely conclude from the above facts, that the indiſcriminate dilatation of gun-ſhot wounds is not a meaſure of neceſſity. I will even add, that it often gives riſe to pain, inflammation, and many troubleſome ſymptoms, which are not merely the effects of the wound. I ſhall mention an obſervation in this place, which I have often ſeen verified, and which I ſhould ſuppoſe can ſcarcely be overlooked by any one, viz. that the pain and inflammation are greater, and conſequently that the cure is ſlower in ſimple fleſh wounds, where the ſurgeon has recourſe to dilatation, dilatation, than where the knife is not employed. This perhaps will be reckoned a ſmall inconvenience; but I will add farther, that the effects of dilatation are peculiarly pernicious in wounds of the joints. It is well known that the acceſs of air, which is favoured by an enlargement of the orifice, is particularly hurtful in wounds which penetrate into cavities. In the joints, after being thus expoſed, the growth of fungous excreſcences is ſcarcely to be reſtrained, and anchyloſis is the leaſt bad effect to be expected.

But though it appears that the dilatation of gun-ſhot wounds is hurtful or ſuperfluous in the caſes which I have mentioned, there are certainly others in which it is both neceſſary and proper. If, for inſtance, a ball, or the fragment of a bone, can be extracted by means of an enlargement of the orifice, no perſon will pretend to ſay that ſuch enlargement ought not to be made. It is likewiſe proper, where wounds run ſuperficially under the ſkin, that they be laid open through the whole of their length; and in many caſes where inflammation comes upon a wounded limb, the muſcles of which are covered by a tendinous expanſion, and, as it were, girded by it, a deep and free dilatation dilatation is then of obvious and great fervice. Such dilatations, however, ought to be deferred till the occafion requires them.

Such, as far as my obfervation goes, are the advantages and difadvantages of dilating gun-fhot wounds. There are alfo other practices employed by furgeons, which, inftead of being productive of benefit, often occafion no fmall fhare of harm. It appears to be an axiom in furgery, that a warm poultice, in the firft ftages of gun-fhot wounds, is an application not to be difpenfed with. I do not deny that in cold climates, where the fibre is tenfe and rigid, they often alleviate pain and promote digeftion; but I am alfo of opinion, that, in the tropical climates, or even in the fouthern provinces of North America, they are not only unneceffary, but fometimes actually the caufe of very troublefome complaints. I have obferved, in numerous inftances, that pain and inflammation were obvioufly increafed by the ufe of warm poultices, and even that fuppurations, independent of the fuppuration of the wounds, were fometimes produced merely from the heat and relaxation which that application occafioned. Befides poultices are inconvenient and unpleafant in warm weather, and may be reckoned koned koned among the caufes which promote the generation of maggots. I might, therefore, I believe, fafely advife, that the practice be difcontinued in the above-mentioned climates. I might even add, that I am fufficiently warranted to recommend a contrary one. Thus I have often found benefit from the application of bandages wet with laudanum or fpirituous liquors, and, above all, from the pouring of cold water upon the wounded limb. The good effects of thefe, in difpofing the wounds to heal, were very remarkable.

To the above obfervations I fhall beg leave to add another, which I believe is not commonly attended to. Reft and quiet are uniformly fuppofed to be proper in the treatment of wounds. Where wounds penetrate into the cavities of the body, motion would often be dangerous; and where the legs or thighs are broken, it cannot be attempted unlefs with great caution: but in the ordinary circumftances of flefh wounds the advantages of moving about, even of travelling, or continuing to march, are great and obvious. Not to adduce the American militia only as a proof of this opinion, I fhall mention, that after the action of Guildford, in North Carolina, every man who was capable of being conveyed either in litter, waggon, or on horfeback, was carried with the army. The healing progrefs was rapid while we were upon the march; I imagined that it proceeded more flowly when we halted for a few days at Croffcreek; and when we came to fix our ftation at Wilmington it was, in fome degree, retrograde: but this was probably owing to the foldiers having accefs to fpirituous liquors.

*Stockton,*

October 29, 1790.

